# Hypereosinophilic Syndrome in a Patient With Cystic Fibrosis: A Rare Case of Cardiac Involvement and Response to Mepolizumab

**DOI:** 10.7759/cureus.87264

**Published:** 2025-07-04

**Authors:** Moshe Heching, Tzlil Grinberg, Ashraf Hamdan, Lev Freidkin, Shimon Izhakian, Maayan Huberman Samuel, Joel Weinberg, Mordechai R Kramer

**Affiliations:** 1 Pulmonary Department, Rabin Medical Center, Beilinson Hospital, Petah Tikva, ISR; 2 Cardiology Department, Rabin Medical Center, Beilinson Hospital, Petah Tikva, ISR

**Keywords:** cystic fibrosis, eosinophilia, eosinophilic myocarditis, hypereosinophilic syndrome, mepolizumab, perimyocarditis

## Abstract

Hypereosinophilic syndrome (HES) is a rare condition characterized by persistent eosinophilia (eosinophil count ≥1.5 × 10^9^/L) and end-organ damage in the absence of an identifiable cause. Cardiac involvement is common and may lead to life-threatening complications. Cystic fibrosis (CF) is a chronic multisystem disease predominantly associated with neutrophilic inflammation, and eosinophilic disorders are less often reported in this population. A 32-year-old woman with CF, complicated by CF-related diabetes and pancreatic insufficiency, presented with chest pain and peripheral eosinophilia (3.2 × 10⁹/L); infectious, autoimmune, and allergic evaluations were negative. Imaging revealed perimyocarditis, and systemic corticosteroids were initially effective but discontinued due to cushingoid side effects and anasarca. She subsequently experienced a recurrence of chest pain accompanied by eosinophilia (1.7 × 10⁹/L), and a diagnosis of idiopathic HES was made based on persistent eosinophilia, cardiac involvement, and exclusion of secondary causes. She responded favorably to monthly subcutaneous mepolizumab, a monoclonal antibody that prevents interleukin-5 (IL-5) from binding to its receptor, thereby inhibiting the recruitment and activation of eosinophils, with resolution of eosinophilia and improvement in symptoms. This case underscores the importance of considering HES in CF patients presenting with unexplained eosinophilia and extrapulmonary symptoms. It also illustrates the efficacy of targeted biologic therapy in managing idiopathic HES when corticosteroids are poorly tolerated.

## Introduction

Hypereosinophilic syndrome (HES) comprises a heterogeneous group of rare disorders defined by sustained peripheral eosinophilia (absolute eosinophil count ≥1.5 × 10⁹/L) and eosinophil-mediated end-organ damage or dysfunction in the absence of a discernible primary or secondary cause, including parasitic infections, hematologic malignancies, autoimmune conditions, allergic diseases, and drug reactions [1]. Prevalence estimates vary, with up to one case per 100,000 individuals [2]. Cardiac involvement, including eosinophilic myocarditis and pericarditis, is among the most serious complications, contributing significantly to morbidity and mortality [3]. Advances in biologic therapies, particularly monoclonal antibodies targeting interleukin-5 (IL-5) such as mepolizumab, have improved disease control and reduced corticosteroid dependence in affected patients [4].

Cystic fibrosis (CF) is a multisystem genetic disorder generally affecting the lungs. Due to mutations in the CFTR gene leading to defective chloride transport, people with CF suffer from thickened airway secretions and pulmonary disease, primarily characterized by chronic airway inflammation and bronchiectasis. The typical inflammatory profile in CF is marked by robust neutrophilic infiltration. As such, HES would be less anticipated in this population, as CF airway inflammation is principally neutrophilic rather than eosinophilic. Eosinophilia in CF has often been associated with allergic atopy as well as allergic bronchopulmonary aspergillosis (ABPA), a hypersensitivity reaction to aspergillus. However, emerging evidence from cohort studies and registry data has identified eosinophilic phenotypes (commonly defined as ≥300 cells/μL) in up to 20% of patients with bronchiectasis, questioning the immunologic heterogeneity of CF [5-7]. To date, no reports have described HES in a patient with CF, nor have cardiac manifestations of HES been documented in this population. Here, we present what is, to our knowledge, the first reported case of HES with perimyocarditis in a patient with CF who improved with mepolizumab treatment, highlighting the diagnostic challenge and therapeutic implications.

## Case presentation

A 32-year-old woman with CF (genotype F508del/W1282x), complicated by pancreatic insufficiency and CF-related diabetes, presented to the hospital with acute onset of chest pain and headache, worsening over the course of the prior 10 days. The chest pain was worse on exertion and deep inhalation and radiated to her left shoulder. She denied fever, weakness, or increase in cough or sputum. Physical examination was remarkable only for diffuse crackles on auscultation, cardiac sounds were normal, and no rash or edema was observed. Her maintenance therapy included elexacaftor/tezacaftor/ivacaftor (ETI). Laboratory evaluation in the emergency room (ER) revealed marked peripheral eosinophilia (3.2 × 10⁹/L, 30.8%) and a mild elevation in cardiac troponin (47 → 39 ng/L; reference range: 0-14 ng/L). ECG showed sinus tachycardia with no signs of ischemia. Review of the electronic medical record showed increasing eosinophilia two months prior to presentation (1.6 x 10⁹/L). Coronary computed tomography angiography performed in the ER ruled out coronary disease and pulmonary embolism and demonstrated stable bronchiectatic changes associated with CF unchanged from prior imaging. Head computed tomography (CT) showed no evidence of sinusitis or nasal polyps. She was admitted to the internal medicine ward due to persistent symptoms, where she underwent lumbar puncture and transthoracic echocardiography, which were unremarkable, with an ejection fraction of 65% and no valvular pathologies.

**Figure 1:**
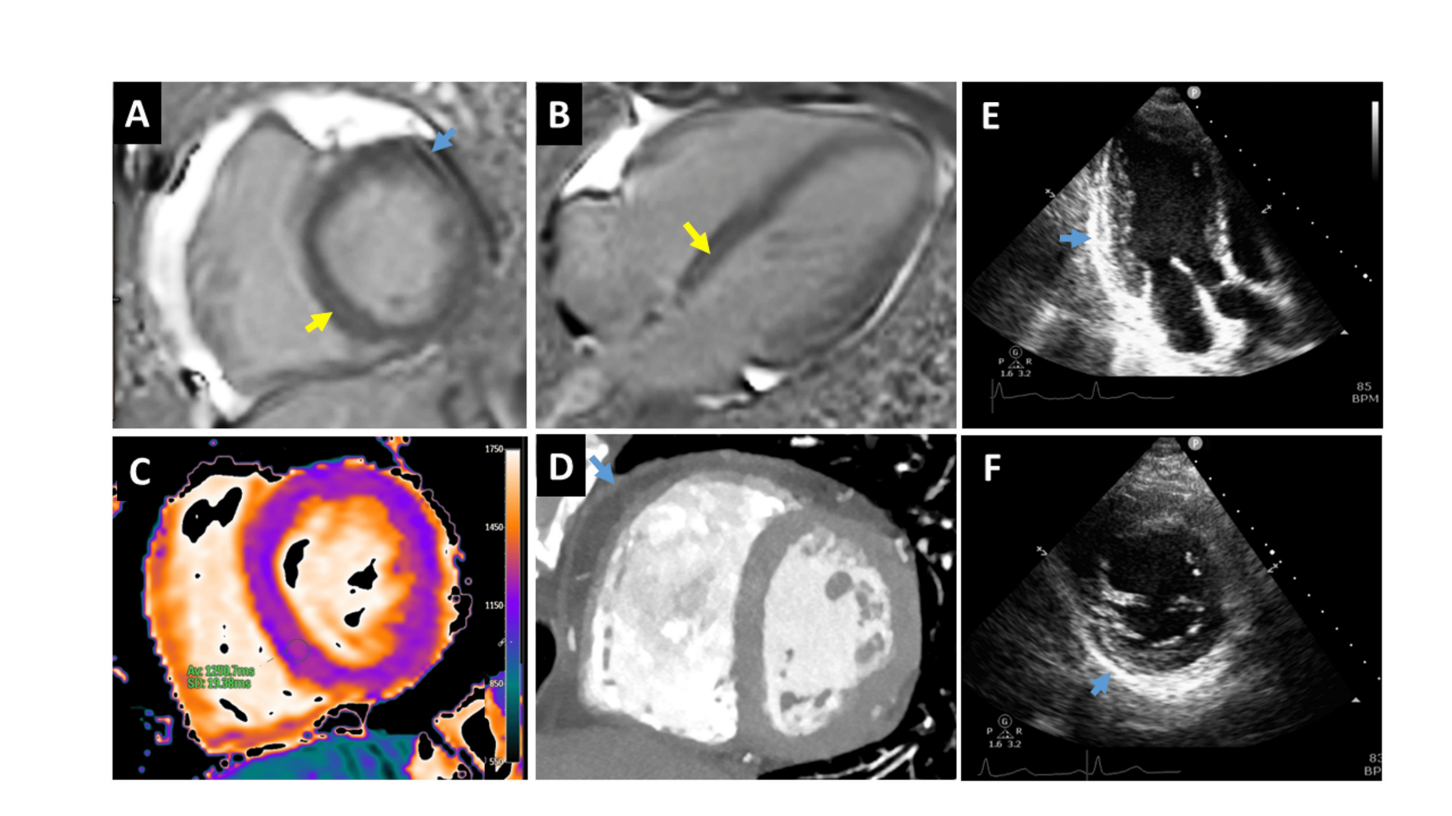
Multimodality images for the diagnosis of perimyocarditis CMR images demonstrate mid-wall late gadolinium enhancement (yellow arrows) in the basal septum (A, B) and diffusely increased T1 values (C) consistent with the diagnosis of myocarditis. CCTA (D) and echo images (E, F) demonstrate pericardial thickening (blue arrows), in addition to late gadolinium enhancement (LGE) of the pericardium (A), consistent with the diagnosis of pericarditis.  Of note, the CMR findings are less pronounced than more fulminant presentations found in the medical literature, which can exhibit subendocardial fibrosis and ventricular thrombi. This case represents an earlier stage presentation with increased T1 values and LGE, which are sufficient for the diagnosis given the clinical context. CCTA, coronary computed tomography angiography; CMR, cardiac magnetic resonance imaging

During the admission, autoimmune and infectious evaluations, including antinuclear antibodies (ANA), antineutrophil cytoplasmic antibodies (ANCA), parasitic serology for strongyloides, stool tests for ova and parasites, total immunoglobulin E (IgE), and Aspergillus fumigatus-specific IgE, were negative or within normal limits. Peripheral blood smear did not identify dysplasia, circulating blasts, or other hematologic abnormalities. Cardiac magnetic resonance imaging (CMR) revealed late gadolinium enhancement of the pericardium and focal myocardial involvement, consistent with perimyocarditis given the clinical context (Figure 1).

The patient was initially treated with high-dose systemic corticosteroids (prednisone 1 mg/kg/day); however, therapy was tapered to discontinue after three weeks due to the development of cushingoid features and anasarca. Prior to discharge, she was transitioned to colchicine (0.5 mg twice daily) to manage the perimyocarditis, as NSAIDs were contraindicated due to a prior nephrotoxic reaction.

Comprehensive outpatient evaluation over the ensuing three months included bronchoscopy for bronchial alveolar lavage, excluding eosinophilic pneumonia and aspergillosis, and upper endoscopy demonstrating no intraepithelial eosinophils, excluding eosinophilic esophagitis. Pulmonary function tests (FEV1 55% and FVC 67%) were unchanged from baseline. Fluorescence in situ hybridization (FISH) results from peripheral blood showed normal interphase cells and no metaphase cells, with no evidence of a translocation at chromosome band q11.2 or the t(9;22)(q34) translocation. The lack of organomegaly, cytopenias, abnormalities in blood count, as well as atypical or dysplastic cells on blood smear, rendered a neoplastic etiology unlikely. Given the low suspicion for clonal disorder due to the normal blood results and negative FISH cytogenetics, a bone marrow biopsy was not performed.

**Figure 2:**
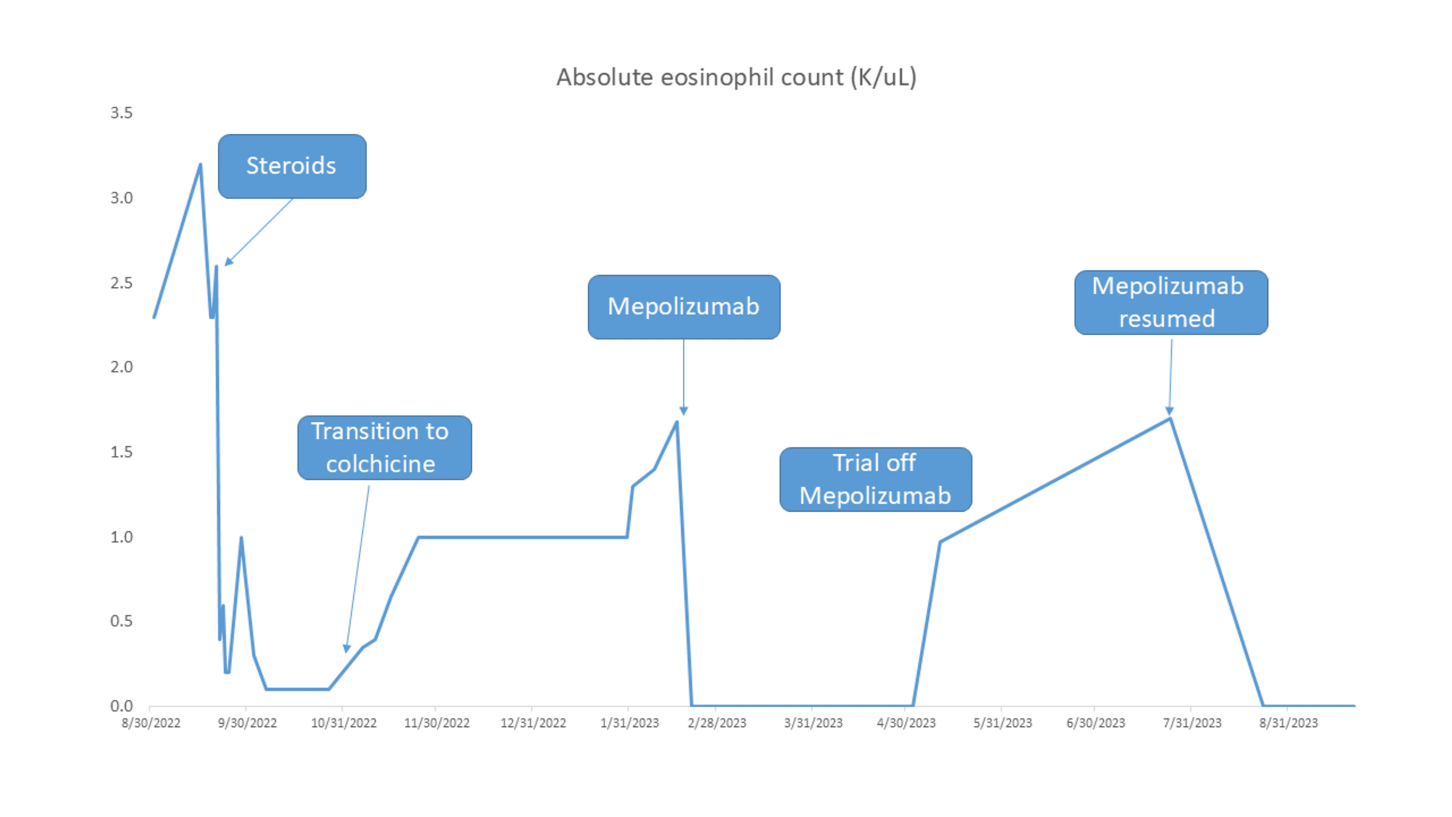
Absolute eosinophil count Peripheral blood eosinophils from the patient's first presentation with chest pain through the ultimate decision to maintain monthly treatment of mepolizumab.

Despite treatment with colchicine, her eosinophil count began to rise, and 3.5 months after discontinuing steroids, she experienced a second episode of chest pain, characterized by eosinophilia (1.68 × 10⁹/L) and mild troponin elevation. Based on the persistence of eosinophilia, evidence of cardiac involvement, and exclusion of secondary causes, a diagnosis of idiopathic HES was made. Subcutaneous mepolizumab was initiated after 4.5 months from presentation, leading to resolution of peripheral eosinophilia and chest pain. The patient did not exhibit any signs of hypersensitivity, localized reactions, or respiratory tract infections attributable to the initiation of mepolizumab. A trial to discontinue mepolizumab after two months of treatment, yet continuing only colchicine for management of pericarditis, led to a third episode of eosinophilia (1.7 × 10⁹/L) and chest pain, after which monthly mepolizumab was resumed (Figure 2).

Subsequent CMR study demonstrated resolution of inflammation and no evidence of fibrosis. A mild troponin elevation persisted, but the patient remained clinically stable over 12 months of follow-up on mepolizumab.

## Discussion

HES refers to a heterogenous group of rare disorders characterized by persistent peripheral eosinophilia (absolute eosinophil count ≥1.5 × 10⁹/L) and eosinophil-mediated end-organ damage or dysfunction in the absence of a discernible primary or secondary cause [1]. Although rare, with a prevalence estimated at fewer than 1 in 100,000 individuals, HES can present with a wide range of clinical manifestations [2]. Commonly affected systems include the heart, lungs, skin, gastrointestinal tract, and peripheral nervous system [1].

Diagnosis of HES requires confirmation of sustained eosinophilia (persistent blood eosinophilia ≥1.5 × 10⁹/L and ≥10% eosinophils) over the course of two to four weeks, evidence of organ damage or dysfunction attributable to the eosinophilia, and exclusion of primary and secondary etiologies including parasitic infections, hematologic malignancies, autoimmune conditions, allergic diseases, and drug reactions [1,8]. The absence of any end-organ damage and other identifiable primary or secondary etiology would militate for idiopathic eosinophilia, a diagnosis of exclusion [9]. Diagnostic evaluation should be guided by clinical presentation and includes a focused history, physical examination, and organ-specific investigations [8]. In a suspected malignancy, further evaluation with bone marrow biopsy, cytogenetic testing, and flow cytometry may be warranted [10]. In this case, the eosinophilia was persistent over the course of more than three months, meeting the requirement for sustained eosinophilia. The absence of systemic signs of infection, negative autoimmune markers, and negative Aspergillus serologies excluded such etiologies and supported the diagnosis of idiopathic HES.

Cardiac involvement, present in up to 20% of patients with HES, is a major contributor to morbidity and mortality [8]. Eosinophilic myocarditis results from eosinophilic infiltration of myocardial tissue, leading to inflammation, necrosis, and fibrosis [11]. Idiopathic eosinophilic myocarditis involves isolated cardiac eosinophilic infiltration without systemic eosinophilia, whereas cardiac involvement in the context of persistent eosinophilia supports the diagnosis of HES [11]. Presentations can range from asymptomatic myocardial injury to fulminant heart failure, arrhythmias, and thromboembolic complications [11]. Mildly elevated troponin levels are suggestive of myocarditis, which can persist even after treatment that alleviates symptoms [11]. Diagnostic imaging, particularly CMR, plays a central role in identifying myocardial involvement and differentiating it from other causes of cardiac injury [12]. In some cases in which CMR results are indeterminate, endomyocardial biopsy may be required to confirm eosinophilic infiltration [13].

Our patient presented with perimyocarditis confirmed on CMR in the context of unexplained eosinophilia. While eosinophilic myocarditis is a recognized complication of HES, it can be found in other eosinophil-associated disorders, specifically eosinophilic granulomatosis with polyangiitis (EGPA). Our patient was ANCA-negative; however, EGPA can be diagnosed based on clinical features despite a negative ANCA. However, the absence of characteristic EGPA features (e.g., asthma, nasal polyps, sinusitis, new pulmonary infiltrates, peripheral neuropathy or hematuria) made EGPA unlikely [14]. Drug reaction with eosinophilia and systemic symptoms (DRESS) was also considered, but our patient had no features of systemic hypersensitivity. Drug-induced eosinophilia generally occurs within the first few weeks of exposure, although onset can be delayed for months [15]. Our patient had been initiated on ETI over 12 months prior to the eosinophilia, and importantly ETI has not been associated with eosinophilia or DRESS.

Although CF is predominantly associated with neutrophilic airway inflammation, peripheral eosinophilia may occur, even in the context of eosinophilic pneumonia or ABPA [16]. Recent studies have identified an eosinophilic endotype in up to 20% of patients with non-CF bronchiectasis, often associated with more severe disease and frequent exacerbations [5-6,17]. A subset of patients with CF exhibit an eosinophilic endotype characterized by elevated eosinophils, increased comorbidities, and a higher burden of complications [18]. As such, the presence of eosinophilia in a CF patient can reflect a bronchiectasis endotype or ABPA. However, in the present case, the eosinophilia could not be attributed to ABPA due to a lack of elevated IgE or characteristic imaging findings. Furthermore, the presence of cardiac (extra-pulmonary) involvement would not be consistent with eosinophilic bronchiectasis and, therefore, supported the diagnosis of idiopathic HES. To our knowledge, this is the first reported case of HES in a patient with CF.

Treatment of HES aims to reduce eosinophil-mediated tissue damage and prevent recurrence. First-line therapy typically includes systemic corticosteroids, particularly in acute or life-threatening situations [1]. However, in this case, corticosteroids were discontinued due to the development of cushingoid features and anasarca. CF patients are particularly susceptible to corticosteroid-related complications, including iatrogenic Cushing’s syndrome, adrenal suppression, fluid retention, and metabolic disturbances [19-20]. Alternative therapies for HES include hydroxyurea, interferon-α, and biologics targeting the IL-5 axis [1].

Mepolizumab, a monoclonal antibody targeting IL-5, has demonstrated efficacy in reducing eosinophil counts and preventing disease flares in patients with HES [21]. Mepolizumab prevents IL-5 from binding to its receptor, thereby inhibiting the recruitment and activation of eosinophils, leading to resolution of eosinophilia and improvement in symptoms. Clinical trials and meta-analyses have confirmed its benefit, particularly in patients with pulmonary or single-organ involvement [22]. Mepolizumab led to a resolution of eosinophilia and clinical symptoms in this case and was well-tolerated following corticosteroid withdrawal. Of note, benralizumab, an anti-IL-5 receptor monoclonal antibody, is also emerging as an effective treatment option in refractory or corticosteroid-intolerant cases [23]. In this case, mepolizumab was chosen due to regulatory guidelines and medical insurance coverage.

Management of HES over the long-term generally requires continuation of an effective treatment regimen, monitoring for disease activity and organ involvement, and minimizing treatment-related adverse effects. With respect to mepolizumab treatment, one study showed that upon achieving disease remission, reducing the monthly dosage of mepolizumab from 300 mg to 100 mg can effectively maintain remission of the disease [24]. In this case, our patient subsequently independently reduced her dosage to 200 mg monthly due to headaches, which she attributed to the higher 300 mg dose, with no adverse effects or flareups.

## Conclusions

This report describes the first documented case of HES with perimyocarditis in a patient with CF. This case underscores the importance of considering HES in patients with CF who present with unexplained eosinophilia and extrapulmonary symptoms. While eosinophilic myocarditis has been well-documented in other eosinophilic conditions, this is, to our knowledge, the first report linking HES with perimyocarditis in a CF patient. It emphasizes the need to broaden differential diagnoses in CF patients presenting with unexplained eosinophilia and cardiac symptoms. The patient’s positive response to mepolizumab demonstrates the potential utility of targeted biologic therapy in managing idiopathic HES when corticosteroids are contraindicated or poorly tolerated and its potential as an effective long-term management to achieve disease remission. Future research should address the appropriate indications for biologic therapy in HES, in terms of end-organ involvement, treatment rationale, and clinical presentation - whether CF, non-CF bronchiectasis, or other pulmonary disorders. In addition, further clinical data are required to better assess the potential for individualized dosing, as well as optimizing long-term management strategies to maximize the clinical benefit of biologic therapy in HES.
